# DPD Quantification in Cardiac Amyloidosis: A Novel Imaging Biomarker

**DOI:** 10.1016/j.jcmg.2020.03.020

**Published:** 2020-06

**Authors:** Paul R. Scully, Elizabeth Morris, Kush P. Patel, Thomas A. Treibel, Maria Burniston, Ernst Klotz, James D. Newton, Nikant Sabharwal, Andrew Kelion, Charlotte Manisty, Simon Kennon, Muhiddin Ozkor, Michael Mullen, Neil Hartman, Perry M. Elliott, Francesca Pugliese, Philip N. Hawkins, James C. Moon, Leon J. Menezes

**Affiliations:** a Barts Heart Centre, St. Bartholomew’s Hospital, London, United Kingdom; b Institute of Cardiovascular Sciences, University College London, London, United Kingdom; c Clinical Physics, St. Bartholomew’s Hospital, London, United Kingdom; d Siemens Healthineers, Forchheim, Germany; e John Radcliffe Hospital, Oxford University Hospitals, Oxford, United Kingdom; f Nuclear Medicine, Abertawe Bro Morgannwg University HB, Swansea, United Kingdom; g William Harvey Research Institute, Queen Mary University of London, London, United Kingdom; h NIHR Barts Biomedical Research Centre, London, United Kingdom; i National Amyloidosis Centre, University College London, London, United Kingdom; j Institute of Nuclear Medicine, University College London, London, United Kingdom; k NIHR University College London Hospitals Biomedical Research Centre, London, United Kingdom

**Keywords:** cardiac amyloidosis, DPD scintigraphy, SPECT/CT quantification, AL, amyloidosis, primary light-chain amyloidosis, ATTR, transthyretin-related amyloidosis, ATTR-CA, transthyretin-related cardiac amyloidosis, CI, confidence interval, CT, computed tomography, DPD, ^99m^Tc-3,3-diphosphono-1,2-propanodicarboxylic acid, ECV_CT_, extracellular volume quantification by computed tomography, H/CL ratio, heart to contralateral lung ratio, PYP, ^99m^Tc-pyrophosphate, ROI, region of interest, SPECT, single-photon emission computed tomography, SUV, standardized uptake value, VOI, volume of interest

## Abstract

**Objectives:**

To assess whether single-photon emission computed tomography (SPECT/CT) quantification of bone scintigraphy would improve diagnostic accuracy and offer a means of quantifying amyloid burden.

**Background:**

Transthyretin-related cardiac amyloidosis is common and can be diagnosed noninvasively using bone scintigraphy; interpretation, however, relies on planar images. SPECT/CT imaging offers 3-dimensional visualization.

**Methods:**

This was a single-center, retrospective analysis of ^99m^Tc-3,3-diphosphono-1,2-propanodicarboxylic acid (DPD) scans reported using the Perugini grading system (0 = negative; 1 to 3 = increasingly positive). Conventional planar quantification techniques (heart/contralateral lung, and heart/whole-body retention ratios) were performed. Heart, adjacent vertebra, paraspinal muscle and liver peak standardized uptake values (SUV_peak_) were recorded from SPECT/CT acquisitions. An SUV retention index was also calculated: (cardiac SUV_peak_/vertebral SUV_peak_) × paraspinal muscle SUV_peak_. In a subgroup of patients, SPECT/CT quantification was compared with myocardial extracellular volume quantification by CT imaging (ECV_CT_).

**Results:**

A total of 100 DPD scans were analyzed (patient age 84 ± 9 years; 52% male): 40 were Perugini grade 0, 12 were grade 1, 41 were grade 2, and 7 were grade 3. Cardiac SUV_peak_ increased from grade 0 to grade 2; however, it plateaued between grades 2 and 3 (p < 0.001). Paraspinal muscle SUV_peak_ increased with grade (p < 0.001), whereas vertebral SUV_peak_ decreased (p < 0.001). The composite parameter of SUV retention index overcame the plateauing of the cardiac SUV_peak_ and increased across all grades (p < 0.001). Cardiac SUV_peak_ correlated well (r^2^ = 0.73; p < 0.001) with ECV_CT_. Both the cardiac SUV_peak_ and SUV retention index had excellent diagnostic accuracy (area under the curve [AUC]: 0.999). The heart to contralateral lung ratio performed the best of the planar quantification techniques (AUC: 0.987).

**Conclusions:**

SPECT/CT quantification in DPD scintigraphy is possible and outperforms planar quantification techniques. Differentiation of Perugini grade 2 or 3 is confounded by soft tissue uptake, which can be overcome by a composite SUV retention index. This index can help in the diagnosis of cardiac amyloidosis and may offer a means of monitoring response to therapy.

Amyloidosis is a multisystem condition characterized by the extracellular deposition of abnormally folded protein fibrils, which result in progressive organ dysfunction (1). Primary light chain (AL) and transthyretin-related amyloidosis (ATTR) commonly affect the heart; the latter can either be associated with a TTR gene mutation (variant ATTR) or not (wild-type ATTR). Previously believed to be rare, more recent research has identified cardiac ATTR (ATTR-CA) in a significant proportion (14% to 16%) of elderly patients with aortic stenosis (2, 3), as well as in 13% of the population with heart failure with preserved ejection fraction (4). A primary driver of this realization is the development of noninvasive diagnostic techniques, which reduce the need for endomyocardial biopsy in an often frail and comorbid population. A key technique in this regard is bone scintigraphy (^99m^Tc-3,3-diphosphono-1,2-propanodicarboxylic acid [DPD], ^99m^Tc-pyrophosphate [PYP], and ^99m^Tc-hydroxymethylene diphosphonate), which, coupled with the exclusion of plasma cell dyscrasia, now offer a noninvasive diagnosis for ATTR-CA (5).

DPD scintigraphy is currently reported by using the Perugini grading system, which is a visual score of the delayed (3-h) planar image, graded from 0 (negative) to 3 (strongly positive) (6). This grading system offers little prognostic significance (7). Difficulty can also ensue in differentiating very subtle cardiac uptake (i.e., a Perugini grade 1) from a negative scan with some residual blood pool activity, despite the additional use of single-photon emission computed tomography (SPECT) imaging. The clinical importance of this distinction remains to be determined; however, it may well prove relevant given the new armamentarium of amyloid-specific therapies in development (8, 9, 10). A semi-quantitative technique was also proposed by Perugini et al. (6) that involved using the early and late planar images to calculate heart and whole-body retention, as well as a heart/whole-body ratio.

PYP scintigraphy is an alternative radiotracer used in the United States for the detection of ATTR-CA, with imaging currently recommended at 1-h post-injection with both SPECT and planar acquisitions and optional 3-h SPECT or planar imaging (11). It is generally reported using both a visual grading system and a semi-quantitative heart to contralateral lung (H/CL) ratio from the planar images, with ratios ≥1.5 at 1 h classified as ATTR-positive (11,12). Furthermore, a H/CL ratio ≥1.6 in patients with ATTR-CA seems to predict a worse outcome (13).

By comparison, SPECT allows the three-dimensional visualization of radioactivity within the body, which can be used to display a standardized uptake value (SUV), a semi-quantitative representation of the concentration of radiopharmaceutical in the respective tissues. SPECT quantification has been used in dementia imaging (14) and tumor dosimetry in radioimmunotherapy (15).

Cardiac amyloid deposition increases myocardial extracellular volume (ECV) greater than any other nonischemic cardiomyopathy (16), due to the extracellular deposition of the amyloid fibrils. These increases are detectable using computed tomography imaging (17), which has been validated against both cardiovascular magnetic resonance (18) and invasive biopsy (19).

The goal of the current study was to investigate whether SPECT/computed tomography (CT)–derived SUV quantification would improve DPD diagnostic accuracy and offer a means of quantifying amyloid burden.

## Methods

This work forms part of ATTRact-AS (A Study Investigating the Role of Occult Cardiac Amyloid in the Elderly With Aortic Stenosis; NCT03029026). The relevant local ethics (London–City Road and Hampstead Research Ethics Committee; reference 10/H0721/79) and local site approvals were obtained for those scans performed as part of ATTRact-AS. Quantification of scans performed clinically outside of ATTRact-AS (n = 39) was approved locally as a quality improvement project at Barts Heart Centre (ID 9924). Diagnostic work-up was performed at the National Amyloidosis Centre in the majority of patients (70%).

### DPD scintigraphy

All DPD scans were performed by using either a hybrid SPECT/CT gamma camera (BrightView, Philips Healthcare, Amsterdam, the Netherlands) or a SPECT gamma camera (Symbia, Siemens Healthineers, Erlangen, Germany) following the injection of ∼700 MBq DPD.

The imaging protocol involved an early (5 min) and late (3 h) planar whole-body image, with a SPECT/CT or SPECT only scan of the chest at 3 h. If a CT scan was not performed in the same sitting, then a contemporary CT scan of the chest was used for attenuation correction and SUV analysis (n = 9 patients). DPD scans were reported by 2 experienced clinicians using the Perugini grading system (6), with grade 0 being negative and grades 1 to 3 increasingly positive.

Planar whole-body scans were performed at a scan speed of 20 cm/min; the matrix size was 256 × 1,024 on the Siemens Symbia and 512 × 1,024 on the Philips BrightView. SPECT acquisitions used a contoured orbit with 120 views in a 360° orbit, with 20 s per view and a matrix size of 128 × 128. CT acquisitions of the chest (performed as part of the SPECT/CT imaging) were low dose, ungated, free-breathing, and noncontrast.

### SUV quantification

**Figure 1:**
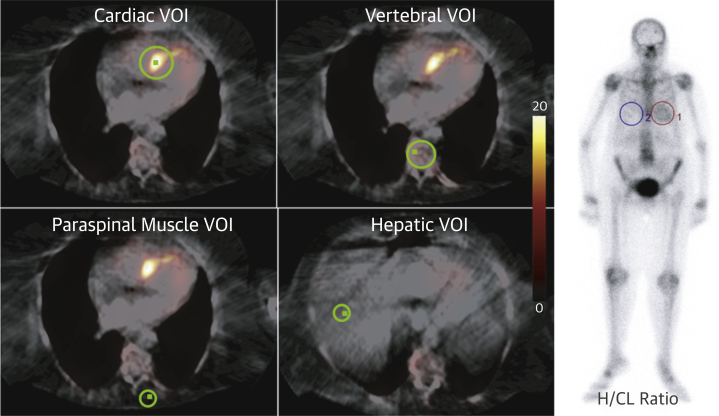
SPECT/CT and H/CL Ratio Quantification Fused axial single-photon emission computed tomography/computed tomography (SPECT/CT) ^99m^Tc-3,3-diphosphono-1,2-propanodicarboxylic acid images demonstrating volume of interest (VOI) **(green)** positioning for standardized uptake value (SUV) quantification on the left. The **small green square** within each VOI represents the peak SUV (SUV_peak_), which enables the reporter to ensure that the recorded SUV_peak_ lies within the desired organ/tissue. On the **right**, 3-h planar, whole-body ^99m^Tc-3,3-diphosphono-1,2-propanodicarboxylic acid image of a different patient with regions of interest over the heart (1) and contralateral lung (4). H/CL = heart/contralateral lung.

SPECT/CT acquisitions of the chest were reconstructed by using Hybrid Recon (Hermes Medical Solutions, Stockholm, Sweden). Injected dose, residual dose, height, weight, and timing data were inputted, and recorded counts per voxel were converted into activity per unit volume and then displayed as an SUV (a parameter representing the concentration of the radiopharmaceutical in the respective tissue). Three-dimensional volumes of interest (VOIs) were placed over the heart, adjacent vertebra, paraspinal muscle, and liver, and peak SUV was recorded (Figure 1). Peak SUV is the highest average SUV within a 1 cm^3^ volume. Care was taken to avoid severe degenerative tracer uptake in the vertebra when placing the relevant VOI.

The exploratory composite parameter of SUV retention index was calculated as: (cardiac peak SUV/vertebral peak SUV) × paraspinal muscle peak SUV.

### Conventional planar quantification

Heart and whole-body retention, as well as heart/whole-body ratio were calculated by comparing regions of interest (ROIs) placed over the heart, kidneys, and bladder on the early and late planar DPD images, as described by Perugini et al. (6).

### H/CL Ratio quantification

A two-dimensional ROI was placed over the heart and then mirrored onto the contralateral lung on the late anterior whole-body planar images (acquired 3 h post-DPD tracer injection) (Figure 1). Mean counts per pixel were recorded for each ROI. As per the American Society of Nuclear Cardiology practice points for PYP scintigraphy (11), the H/CL ratio was calculated as the mean counts per pixel in the cardiac ROI divided by the same in the contralateral lung ROI.

### Extracellular volume quantification by CT

All CT scans were performed on a Somatom FORCE scanner (Siemens Healthineers, Erlangen, Germany). The technique for ECV quantification has been described elsewhere (20); additional pre-contrast and 3-min post-contrast datasets were acquired. These datasets were averaged, subtracted to provide a partition coefficient, and then co-registered with the CT coronary angiogram images. The patient’s hematocrit measurement (usually taken on the same day) was inputted, and the myocardial ECV was calculated as follows: ECV_CT_ = (1 − hematocrit) × (ΔHU_myo_ /ΔHU_blood_), where ΔHU is the change in Hounsfield unit attenuation pre- and post-contrast (i.e., HU_post-contrast_ − HU_pre-contrast_) (17, 18, 19, 20, 21).

### Statistical analysis

Statistical analysis was performed using IBM SPSS Statistics version 25 (IBM SPSS Statistics, IBM Corporation, Armonk, New York). Where appropriate, results are described as mean ± SD or median (interquartile range). Kruskal-Wallis analysis of variance was used when comparing more than 2 groups as the omnibus test, with the Dunn-Bonferroni test for pairwise comparison. Receiver-operating characteristic curve analysis was performed to assess diagnostic performance. The Fisher exact test was used for categorical data, Spearman’s rank-order correlation was used to assess correlation, and the DeLong test was used to compare areas under the curves. A 2-sided p value <0.05 was considered statistically significant.

## Results

Analysis was performed on the DPD scans of 100 patients (mean age 84 ± 9 years; 52% male), of which forty were grade 0, twelve were grade 1, forty-one were grade 2, and seven were grade 3. All patients were identified retrospectively and selected for their DPD result; therefore, this is not a prevalence study. The average injected dose was 731 ± 26 MBq. There was a higher proportion of male patients in the DPD-positive cohorts, likely reflecting referral bias.

Thirty-four patients with a positive DPD scan were diagnosed with likely wild-type ATTR, 16 with variant ATTR (thirteen V122I, one ApoA1, one E89Q, and one I107V), and three with AL amyloid (the remaining seven patients either declined or were awaiting further investigation). TTR genotyping was available for 40 (70%) of the 57 patients with suspected ATTR cardiac amyloidosis. Two out of three patients with AL amyloid had a grade 1 DPD scan.

### Perugini planar quantification

Heart, whole-body retention, and heart/whole-body ratio increased with increasing DPD grade (p < 0.001 for trend) (Table 1). Pairwise comparison revealed a significant difference between grades 0 and 2 and between grades 0 and 3 only for all of these parameters.

**Table 1:** Summary of Basic Patient Demographics, With a Breakdown of SUV_peak_, Conventional Planar Quantification, and Heart/CL Ratio Results by DPD Perugini Grade

	Grade 0 (n = 40)	Grade 1 (n = 12)	Grade 2 (n = 41)	Grade 3 (n = 7)	p Value
Demographic characteristics					
Male	12 (30)	9 (75)	27 (66)	4 (57)	**0.003**
Age	86 ± 5	83 ± 12	82 ± 10	80 ± 8	0.11
Amyloid type∗					
Likely wild-type ATTR	–	9 (82)	24 (66)	1 (17)	**0.03**
Variant ATTR	–	0 (0)	11 (31)	5 (83)	**0.001**
AL amyloid	–	2 (18)	1 (3)	0 (0)	0.14
SUV_peak_					
Cardiac	1.0 ± 0.4	3.7 ± 1.5	11.9 ± 3.8	10.6 ± 1.5	**<0.001**
Paraspinal	0.6 ± 0.1	0.9 ± 0.2	1.0 ± 0.3	1.3 ± 0.3	**<0.001**
Vertebral	8.4 ± 1.5	7.2 ± 1.2	6.2 ± 1.9	4.6 ± 0.17	**<0.001**
Hepatic	0.6 ± 0.2	0.6 ± 0.2	0.6 ± 0.3	0.5 ± 0.2	0.87
SUV retention index	0.07 ± 0.03	0.48 ± 0.28	2.04 ± 0.82	3.24 ± 1.04	**<0.001**
Conventional planar					
Heart retention	74.5 ± 7.8	81.2 ± 6.6	83.2 ± 7.0	90.3 ± 4.6	**<0.001**
WB retention	3.6 ± 0.8	4.6 ± 0.9	6.0 ± 1.4	6.7 ± 0.9	**<0.001**
Heart/WB ratio	4.9 ± 0.9	5.7 ± 0.9	7.2 ± 1.6	7.4 ± 1.1	**<0.001**
Heart/CL lung H/CL ratio	1.01 ± 0.10	1.35 ± 0.21	2.23 ± 0.55	2.12 ± 0.59	**<0.001**

Values are n (%) or mean ± SD.

H/CL = heart/contralateral lung; DPD = ^99m^Tc-3,3-diphosphono-1,2-propanodicarboxylic acid; SUV_peak_ = peak standardized uptake value; WB = whole-body.

∗ 7 patients were excluded due to no diagnostic work-up results being available at the time of submission; percentages quoted reflect this. Transthyretin genotyping was available in 70% of the transthyretin-related cardiac amyloidosis (ATTR-CA) population. **Bold** values p value <0.05.

### H/CL ratio

The H/CL ratio increased from grade 0 to grade 2 and then plateaued between grades 2 and 3 (p < 0.001 for trend). Pairwise comparison revealed a significant difference between all grades, except between grades 1 and 3 (p = 0.410) and grades 2 and 3 (p = 1.000).

There was no significant difference in H/CL ratio in those patients with a grade 1 DPD scan between the 2 patients with cardiac AL versus those with ATTR (p = 0.83).

### SPECT/CT quantification

**Figure 2:**
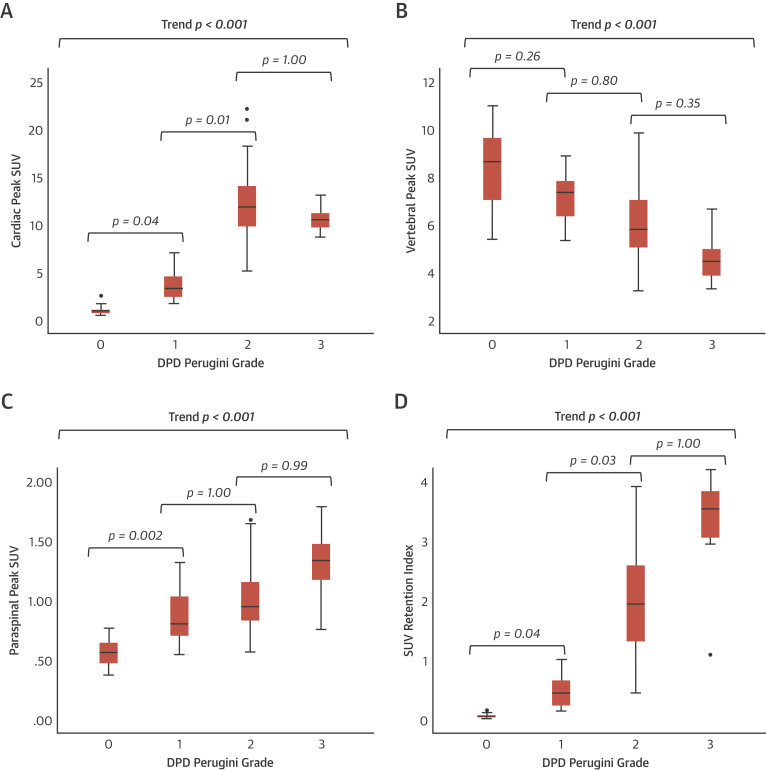
Trends in SPECT/CT Quantification by DPD Perugini Grade **Box and whisker plots** illustrating the trend seen with increasing ^99m^Tc-3,3-diphosphono-1,2-propanodicarboxylic acid (DPD) Perugini grade in SUV_peak_ in the heart **(A)**, vertebra **(B)**, paraspinal muscle **(C)**, and the composite SUV retention index (**D**). Whisker lengths extend to 1.5 times the box height, or if no case has a value in that range, to the minimum and maximum. **Dots** represent outliers. Abbreviations as in Figure 1.

Cardiac peak SUV increased from DPD Perugini grade 0 to grade 2; however, it plateaued between grades 2 and 3 (p < 0.001 for trend) (Table 1, Figure 2). Pairwise comparison revealed a significant difference between all grades except 1 and 3 (p = 0.460) and 2 and 3 (p = 1.000). Paraspinal muscle peak SUV increased from grade 0 to 3 (p < 0.001 for trend), whereas vertebral peak SUV did the opposite (p < 0.001 for trend). There was no difference in hepatic peak SUV between grades (p = 0.870 for trend).

The composite parameter of SUV retention index helped overcome the plateauing of the cardiac peak SUV between grades 2 and 3, increasing across all grades (p < 0.001 for trend) (Table 1, Figure 2). Pairwise comparison showed a significant difference between all grades except 2 and 3 (p = 1.000).

There was no significant difference between the 2 patients with cardiac AL and a grade 1 DPD scan and those with ATTR in the same group in terms of cardiac peak SUV (p = 0.260) or SUV retention index (p = 0.970).

### Comparison between planar and SPECT/CT quantification

**Figure 3:**
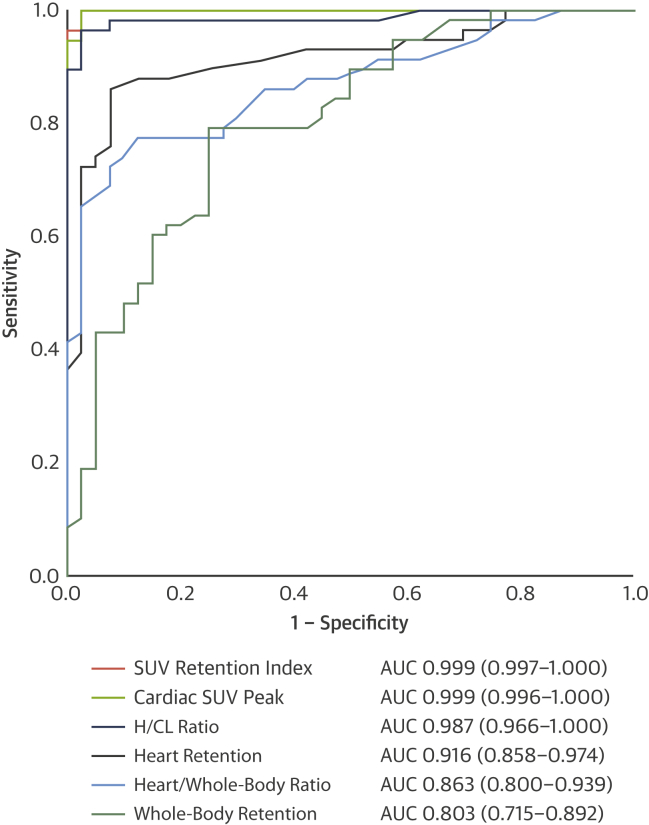
Diagnostic Accuracy for the Detection of Cardiac Amyloid Receiver-operating characteristic curves for the detection of cardiac amyloidosis using the different methods of quantification of ^99m^Tc-3,3-diphosphono-1,2-propanodicarboxylic acid scintigraphy. **Values in parentheses** represent 95% confidence intervals. AUC = area under the curve; other abbreviations as in Figure 1.

The diagnostic accuracy for the detection of any cardiac amyloid was excellent for cardiac peak SUV, with an AUC of 0.999 (0.996 to 1.000), with a cutoff of >1.7 giving a sensitivity of 100% and a specificity of 75% (Figure 3). The SUV retention index performed similarly well with an AUC of 0.999 (0.997 to 1.000), and a cutoff of >0.14 provided the same sensitivity and specificity (p = 0.480 for comparison with cardiac SUV peak).

In terms of planar quantification techniques, the H/CL ratio performed the best with an AUC of 0.987 (0.966 to 1.000), with a ratio >0.97 giving a sensitivity of 100% but a specificity of only 38% (p = 0.270 and 0.250 for comparison with cardiac peak SUV and SUV retention index, respectively). The conventional heart and whole-body retention, as well as the heart/whole-body ratio, did not perform as well as the cardiac peak SUV, SUV retention index, or H/CL ratio (p < 0.050 for all), with AUCs of 0.916 (0.858 to 0.974), 0.803 (0.715 to 0.892), and 0.869 (0.800 to 0.939) (Figure 3).

### Reproducibility and variability

SPECT/CT quantification was repeated for 10 patients (three Perugini grade 3, two grade 1, three grade 2, and two grade 3) by the same reporter (P.R.S.) in a separate sitting, blinded to the first set of results. In total, 50 parameters were assessed (cardiac, vertebral, hepatic, and paraspinal muscle peak SUV, as well as the SUV retention index for each patient). The mean difference in SUV across all measurements (repeat minus initial measurement) was minimal at 0.01 ± 0.38. Importantly, looking at just the cardiac peak SUV (n = 10 measurements), the mean difference remained small at 0.11 ± 0.37. The intraclass correlation coefficient (two-way, mixed effects model, absolute agreement) for all 50 parameters was excellent at 0.997 (95% CI: 0.994 to 0.998) for single measures.

Comparing the intraobserver variability for H/CL ratio for the same 10 patients, there was minimal mean difference between the results at 0.03 ± 0.11. The intraclass correlation coefficient (2-way, mixed effects model, absolute agreement) was very good at 0.993 (95% CI: 0.972 to 0.998) for single measures.

Interobserver variability was also assessed for SPECT/CT quantification and H/CL ratio in 5 patients by a second reporter (K.P.P.), who was blinded to the initial results. The mean difference in SUV across all measurements (K.P.P. minus P.R.S.; 25 measurements) remained small at –0.17 ± 0.71. For cardiac peak SUV alone (5 measurements) this difference was –0.11 ± 0.20. The intraclass correlation coefficient (2-way, mixed effects model, absolute agreement) for all 25 parameters was excellent at 0.989 (95% CI: 0.975 to 0.995) for single measures.

Looking only at the H/CL ratio for the same 5 patients, the mean difference (K.P.P. minus P.R.S.) was 0.05 ± 0.11. The intraclass correlation coefficient (2-way, mixed effects model, absolute agreement) was excellent at 0.994 (95% CI: 0.954 to 0.999) for single measures.

### Comparison between SPECT/CT quantification and ECV quantification by CT

**Figure 4:**
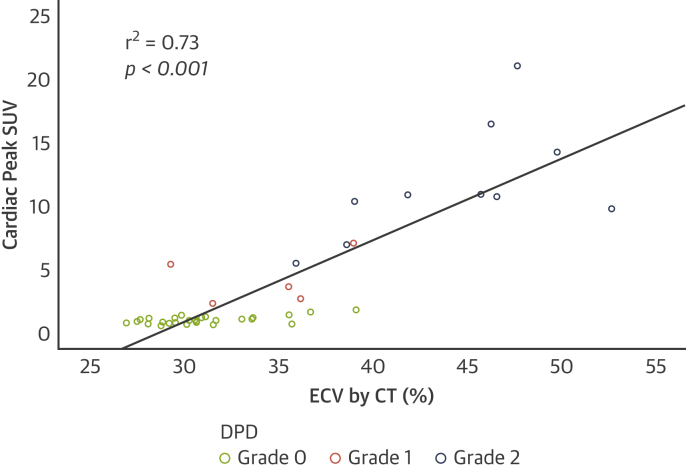
Correlation Between SPECT/CT Quantification and ECV Quantification by CT Scatter plot of cardiac SUV_peak_ against myocardial extracellular volume quantification (ECV) by computed tomography (CT), showing good correlation across DPD Perugini grades 0 to 2. Abbreviations as in Figures 1 and 2.

In a subgroup of patients (n = 44; mean age 87 ± 5 years; 41% male), comparison was made between cardiac peak SUV and ECV_CT_. All patients had severe aortic stenosis: left ventricular ejection fraction 57 ± 8%, peak aortic valve velocity 4.25 ± 0.63 m/s, mean gradient 44 ± 14 mm Hg, and aortic valve area 0.67 ± 0.21 cm^2^. Twenty-nine patients were DPD Perugini grade 0, five were grade 1, and ten were grade 2. Cardiac peak SUV increased across the grades as seen in the overall cohort (p < 0.001 for trend). Myocardial ECV_CT_ increased from 31 ± 3% (grade 0) to 34 ± 4% (grade 1) to 44 ± 5% (grade 2) (p < 0.001 for trend). There was good correlation between the increases in cardiac peak SUV and ECV_CT_ (r^2^ = 0.73; p < 0.001) (Figure 4).

## Discussion

In terms of diagnostic accuracy, SPECT/CT quantification of DPD scintigraphy using either cardiac peak SUV or the composite SUV retention index was exceptional and outperformed planar quantification techniques. It is a simple tool that can help the reporting clinician in making a diagnosis of cardiac amyloidosis, particularly in subtle cases. In centers where SPECT/CT is not available, the H/CL ratio is a viable alternative quantification technique that performs well and with a diagnostic accuracy similar to that seen for the diagnosis of ATTR-CA in the PYP literature (AUC 0.987 vs. 0.960 to 0.992) (12,13). Although well established in PYP reporting, further research is needed to validate the role of the H/CL ratio in DPD (e.g., prognostic thresholds, imaging time points [1 vs. 3 h], how results compare between cardiac AL and ATTR). Conventional planar quantification using heart and whole-body retention, as well as heart/whole-body ratio, did not perform as well; as a result, we have moved away from using these in our clinical practice, obviating the need for the early planar images-improving patient experience and departmental workflow.

**Figure 5:**
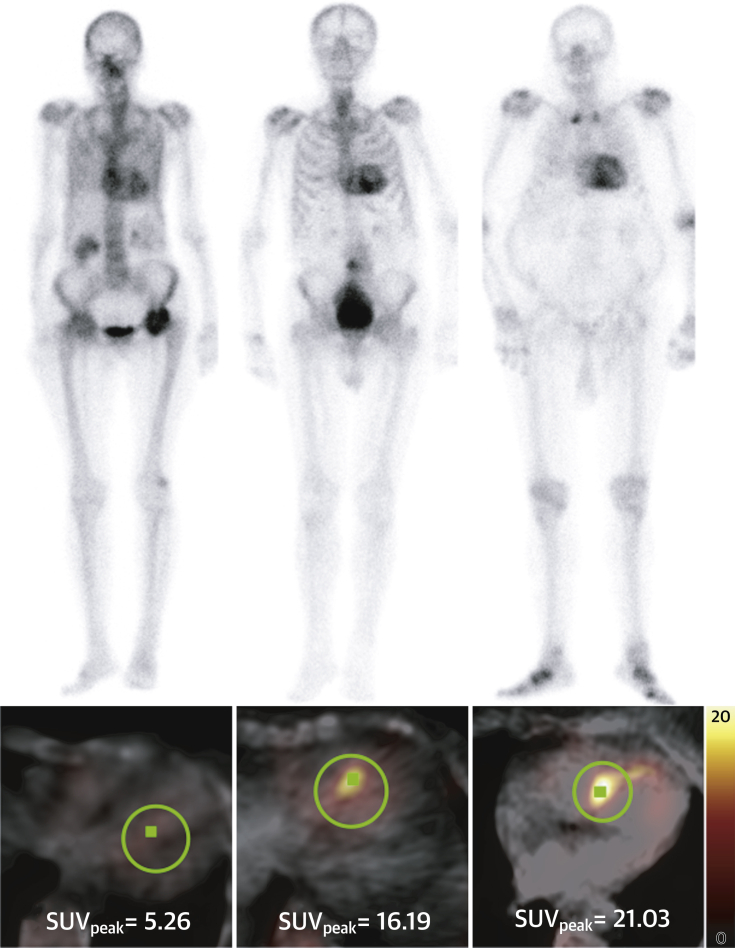
The Unappreciated Spectrum of Disease Planar images from three patients all demonstrating Perugini grade 2 cardiac uptake with some long bone suppression. The cardiac peak standardized uptake value (peak SUV) is markedly different between patients, illustrating the wide range of cardiac involvement that is not apparent or appreciated on planar imaging alone.

In terms of quantifying cardiac amyloid burden, it is apparent that there is a huge spectrum of disease that is not captured in the Perugini grading system (perhaps unsurprising, given there are only 3 grades of positivity used to describe a complex, multisystem disorder) (6). An example of this is the variation in cardiac peak SUV seen between patients with a grade 2 DPD scan, which can range from 5 to 21 (Figure 5). This grading system alone is unlikely to prove sufficiently sensitive to facilitate the monitoring of response (or lack thereof) to amyloid therapies in the future. Unfortunately, what is also apparent is that quantification of cardiac amyloid burden at higher Perugini grades is confounded by competition for the radiotracer from surrounding soft tissue, resulting in little detectable difference between grades 2 and 3 using cardiac peak SUV alone or H/CL ratio. This makes sense given that this increased soft tissue uptake is the reason for the “mild/absent bone uptake” that forms part of the definition of a grade 3 DPD (6). This is further supported by the increasing paraspinal and reducing vertebral peak SUV seen across all grades. This means that a direct measure of cardiac uptake alone is unlikely to prove sufficient, reflecting the complexity of the condition we are dealing with. This being said, it is reassuring that cardiac peak SUV tracked amyloid burden up to grade 2 as measured by ECV_CT_, given that we already know that cardiovascular magnetic resonance–derived ECV can detect disease regression with therapy in cardiac AL amyloid (22) and also carries prognostic significance in both ATTR-CA (23) and cardiac AL amyloid (24). This would suggest that cardiac peak SUV (or, in turn, the derived SUV retention index) may be used to monitor amyloid regression and be associated with prognosis. Further validation is clearly warranted, particularly outside of the elderly population with aortic stenosis studied here, in which the variation in ECV in those without cardiac amyloid is likely to be much less, and therefore the correlation may well prove even better with cardiac peak SUV. Finally, whether these parameters will also track blood biomarkers (e.g., N-terminal pro–B-type natriuretic peptide, troponin T) or predict heart failure hospitalizations is beyond the scope of the current paper but merits investigation.

The composite SUV retention index tries to account for the competition for the DPD from both the vertebra and the paraspinal muscle and perhaps offers a means of better quantifying the amyloid burden, which is otherwise apparent on the planar images (Central Illustration). Further validation in larger, multicenter studies is needed; however, it could serve as a potential imaging biomarker of response to therapy in upcoming and ongoing clinical trials. Further research is also needed on the role it may offer in differentiating cardiac-AL from ATTR-CA, one of the holy grails of cardiac amyloid imaging.

**Central Illustration:**
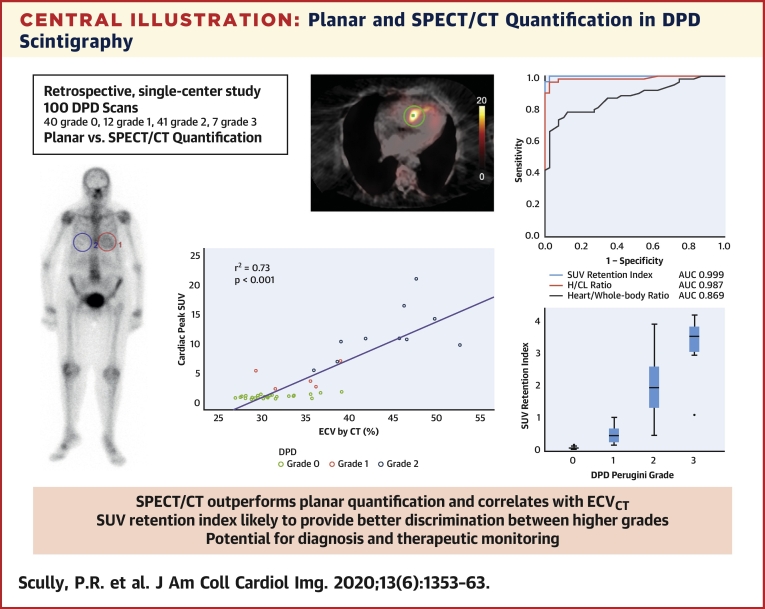
Planar and SPECT/CT Quantification in DPD Scintigraphy Planar and single-photon emission computed tomography/computed tomography (SPECT/CT) quantification was performed retrospectively on 100 ^99m^Tc-3,3-diphosphono-1,2-propanodicarboxylic acid (DPD) scans. SPECT/CT quantification outperformed planar quantification and correlated with extracellular volume quantification by computed tomography (ECV_CT_). Differentiation of Perugini grade 2 and 3 was confounded by soft tissue uptake, which may be overcome by using a composite standardized uptake value (SUV) retention index, providing a potential novel biomarker for monitoring.

### Study limitations

The range of patients reported reflects the clinical practice of a large tertiary cardiac center; however, there were relatively small numbers of DPD Perugini grade 1 and grade 3 patients, which is likely to have effected our results. We also only included 3 patients with AL amyloid, meaning any conclusions drawn primarily relate to the ATTR-CA population. ECV_CT_ was only performed in the subgroup of elderly patients with severe aortic stenosis and, as a result, patients without cardiac amyloid exhibited significant variation in ECV_CT_; this is likely to affect the correlation with cardiac peak SUV, which may well prove even better outside of this population.

## Conclusions

SPECT/CT quantification in DPD scintigraphy is possible and outperforms planar quantification techniques. Differentiation of Perugini grade 2 or 3 is confounded by soft tissue uptake, which can be overcome by using a composite SUV retention index. This index can help in the diagnosis of cardiac amyloidosis and may offer a means of monitoring response to therapy.

Perspectives **COMPETENCY IN MEDICAL KNOWLEDGE:** SPECT/CT quantification of DPD outperforms conventional planar quantification techniques and is a potential tool for diagnosis and therapy monitoring in cardiac amyloidosis.

**TRANSLATIONAL OUTLOOK 1:** SUV retention index is likely to prove a useful imaging biomarker in future trials assessing amyloid-specific therapies.

**TRANSLATIONAL OUTLOOK 2:** Further research is needed to investigate the prognostic significance of the direct measurement of amyloid burden in those undergoing DPD scintigraphy.
