# Correction

**DOI:** 10.1016/j.jcmg.2020.12.001

**Published:** 2021-01

Scully PR, Morris E, Patel KP, Treibel TA, Burniston M, Klotz E, Newton JD, Sabharwal N, Kelion A, Manisty C, Kennon S, Ozkor M, Mullen M, Hartman N, Elliott PM, Pugliese F, Hawkins PN, Moon JC, Menezes LJ

**DPD Quantification in Cardiac Amyloidosis: A Novel Imaging Biomarker**

**J Am Coll Cardiol Img 2020;13:1353-63.**

There was a labeling error in the original conventional planar section of Table 1. The whole-body retention values should read as those ranging from 74.5 ± 7.8 up to 90.3 ± 4.6 across the columns (p < 0.001) and the heart retention values should read as those ranging from 3.6 ± 0.8 up to 6.7 ± 0.9 across the columns (p < 0.001). Please see below for the corrected version of Table 1. The statistics, the interpretation, and the conclusions in the original manuscript remain accurate. The online version of the paper has been corrected to reflect these changes.

**Table 1:** Summary of Basic Patient Demographics, With a Breakdown of SUV_peak_, Conventional Planar Quantification, and Heart/CL Ratio Results by DPD Perugini Grade

	Grade 0 (n = 40)	Grade 1 (n = 12)	Grade 2 (n = 41)	Grade 3 (n = 7)	p Value
Demographic characteristics					
Male	12 (30)	9 (75)	27 (66)	4 (57)	**0.003**
Age	86 ± 5	83 ± 12	82 ± 10	80 ± 8	0.11
Amyloid type∗					
Likely wild-type ATTR	–	9 (82)	24 (66)	1 (17)	**0.03**
Variant ATTR	–	0 (0)	11 (31)	5 (83)	**0.001**
AL amyloid	–	2 (18)	1 (3)	0 (0)	0.14
SUV_peak_					
Cardiac	1.0 ± 0.4	3.7 ± 1.5	11.9 ± 3.8	10.6 ± 1.5	**<0.001**
Paraspinal	0.6 ± 0.1	0.9 ± 0.2	1.0 ± 0.3	1.3 ± 0.3	**<0.001**
Vertebral	8.4 ± 1.5	7.2 ± 1.2	6.2 ± 1.9	4.6 ± 0.1	**<0.001**
Hepatic	0.6 ± 0.2	0.6 ± 0.2	0.6 ± 0.3	0.5 ± 0.2	0.87
SUV retention index	0.07 ± 0.03	0.48 ± 0.28	2.04 ± 0.82	3.24 ± 1.04	**<0.001**
Conventional planar					
WB retention	74.5 ± 7.8	81.2 ± 6.6	83.2 ± 7.0	90.3 ± 4.6	**<0.001**
Heart retention	3.6 ± 0.8	4.6 ± 0.9	6.0 ± 1.4	6.7 ± 0.9	**<0.001**
Heart/WB ratio	4.9 ± 0.9	5.7 ± 0.9	7.2 ± 1.6	7.4 ± 1.1	**<0.001**
H/CL ratio	1.01 ± 0.10	1.35 ± 0.21	2.23 ± 0.55	2.12 ± 0.59	**<0.001**

Values are n (%) or mean ± SD.

H/CL = heart/contralateral lung; DPD = ^99m^Tc-3,3-diphosphono-1,2-propanodicarboxylic acid; SUV_peak_ = peak standardized uptake value; WB = whole-body.

∗ 7 patients were excluded due to no diagnostic work-up results being available at the time of submission; percentages quoted reflect this. Transthyretin genotyping was available in 70% of the transthyretin-related cardiac amyloidosis (ATTR-CA) population. **Bold** values p value <0.05.

The authors apologize for this error.

